# Development of pooled testing system for porcine epidemic diarrhoea using real-time fluorescent reverse-transcription loop-mediated isothermal amplification assay

**DOI:** 10.1186/s12917-018-1498-9

**Published:** 2018-05-29

**Authors:** Thi Ngan Mai, Van Diep Nguyen, Wataru Yamazaki, Tamaki Okabayashi, Shuya Mitoma, Kosuke Notsu, Yuta Sakai, Ryoji Yamaguchi, Junzo Norimine, Satoshi Sekiguchi

**Affiliations:** 1 Animal Infectious Disease and Prevention, Department of Veterinary Sciences, Faculty of Agriculture, University of Miyazaki, Miyazaki, Japan; 2 Faculty of Veterinary Medicine, Vietnam National University of Agriculture, Hanoi, Vietnam; 3 Center for Animal Disease Control, University of Miyazaki, Miyazaki, Japan

**Keywords:** PEDV, RtF-RT-LAMP, One-step RT-PCR, Pooled stool samples

## Abstract

**Background:**

Porcine epidemic diarrhoea (PED) is an emerging disease in pigs that causes massive economic losses in the swine industry, with high mortality in suckling piglets. Early identification of PED virus (PEDV)-infected herd through surveillance or monitoring strategies is necessary for mass control of PED. However, a common working diagnosis system involves identifying PEDV-infected animals individually, which is a costly and time-consuming approach. Given the above information, the thrusts of this study were to develop a real-time fluorescent reverse transcription loop-mediated isothermal amplification (RtF-RT-LAMP) assay and establish a pooled testing system using faecal sample to identify PEDV-infected herd.

**Results:**

In this study, we developed an accurate, rapid, cost-effective, and simple RtF- RT-LAMP assay for detecting the PEDV genome targeting M gene. The pooled testing system using the RtF-RT-LAMP assay was optimized such that a pool of at least 15 individual faecal samples could be analysed.

**Conclusions:**

The developed RtF-RT-LAMP assay in our study could support the design and implementation of large-scaled epidemiological surveys as well as active surveillance and monitoring programs for effective control of PED.

**Electronic supplementary material:**

The online version of this article (10.1186/s12917-018-1498-9) contains supplementary material, which is available to authorized users.

## Background

Porcine epidemic diarrhoea (PED) is caused by PED virus (PEDV), which is characterized by enteritis, vomiting, and watery diarrhoea. This leads to massive economic losses in the swine industry with high mortality in suckling pigs [1]. PED was first observed in England in 1971 and identified in Belgium in 1978 [2, 3]. The disease quickly spread to other European countries such as Belgium, England, Germany, France, and Switzerland in the 1980s, and later to Asian countries including Korea, China, Thailand, and Vietnam [4, 5]. Recently, several epidemics were reported in important swine-producing countries such as USA, Canada, and Japan [6–8].

Control PED programs require effective and rapid surveillance protocols, linked to prompt control procedures, to ensure that epidemics are brought under control quickly. Currently, the identification of infected herd is done by passive surveillance with required reporting of infected herds from veterinarians. However, veterinarians rely on herd demonstrating clinical signs of infection, which can lead to failure to accurately identify PED status and transmission of PEDV to healthy animals. Moreover, surveillance or monitoring is applied to individuals, which is associated with important financial and time obstacles. Therefore, well-designed surveys as well as sensitive, specific, rapid, and simple detection methods are necessary for the identification of infected herd to control PED. Loop-mediated isothermal amplification (LAMP) combines rapidity, simplicity, and high specificity under isothermal conditions [9, 10]. We developed an accurate, timely, and simple real-time fluorescent reverse transcription LAMP (RtF-RT-LAMP) assay (from M gene) using pooled stool samples for PEDV detection. This assay can be applied to strategies for the control, monitoring, and surveillance of PED.

## Methods

### Viruses

PEDV NK94P6 and Fukuoka-1 Tr(−) strains which belong to classical clade (G1) were propagated in Vero-KY5 (Vero) cells. Trypsin was not used to culture PEDV in Vero cells. The NK94P6 and Fukuoka-1 strains were kindly provided by the National Institute of Animal Health, Japan, and the Fukuoka Chuo Livestock Hygiene Service Center, Fukuoka, Japan, respectively. The Vero cells were also provided by the National Institute of Animal Health, Japan. Briefly, Vero cells were cultured in Eagle’s minimal essential medium (EMEM) (Sigma-Aldrich, Tokyo, Japan) supplemented with 10% (*v*/v) foetal bovine serum (FBS) (Funakoshi, Tokyo, Japan), 0.3% (*w*/*v*) tryptose phosphate broth (TPB) (Sigma-Aldrich), and 100 U/ml penicillin-streptomycin (Wako, Tokyo, Japan) at 37 °C in a humidified atmosphere containing 5% CO_2_. Viruses were propagated in Vero cells cultured in EMEM with 2% FBS and 0.3% TPB at 37 °C. The titter of the PEDV NK94P6 and Fukuoka-1 Tr(−) strains were 2.8 × 10^6^ TCID_50_/ml and 2 × 10^4^ TCID_50_/ml, respectively.

Transmissible gastroenteritis coronavirus - TGEV (vaccine strain h-5; Nisseiken, Tokyo, Japan) was propagated in Vero cells; porcine reproductive and respiratory syndrome virus - PRRSV (live PRRS vaccine - Ingelvac PRRS® MLV- Boehringer Ingelheim company); Japanese Encephalitis virus – JEV and Getal virus - GV (live vaccine – Kyoto Biken company, Kyoto, Japan).

### Primers

All primers for RtF-RT-LAMP were designed from the highly conserved M gene sequence of porcine epidemic diarrhoea virus CV777 strain (GenBank accession number: KT323979) using the Primer Explorer 4 (https://primerexplorer.jp/lamp4.0.0/index.html) (Additional file 1). They were synthesized using sequence-grade purification by Hokkaido System Science Co., Ltd. (Sapporo, Japan), which included an outer pair (F3, B3), inner primers (FIP, FIP1, BIP), and a loop pair (loopF, loopB) (Table 1). Nucleotide sequences specific for PEDV were detected by multiple alignments of 997 M gene sequences, available from the DDBJ/EMBL/GenBank database. The one-step RT-PCR used a previously published primer pair on the S gene [11].

**Table 1:** Primers used for RtF-RT-LAMP in this study

Primer	ID	Sequence (5′ to 3′)	Gene location
F3	PED_F3_ID1	TCCTTATGGCTTGCATCAC	25,846–25,864
B3	PED_B3_ID1	CCGTAGACAATTGTTGTAGTGG	26,143–26,122
FIP	PED_FIP_ID1, PED_FIP_ID1modified	GTMGGCCCATCACAGAAGTAGTTTT GGTTGTGGCGCAGGACA	25,983–25,963 (TTTT) 25,903–25,919
BIP	PED_BIP_ID1	CCAACTGGTGTAACGCTAACACTTTTT TACCTGTACGCCAGTAGC	26,010–26,032 (TTTT) 26,087–26,070
LF	PED_LF_ID37	TTTCAGGATTGAAAGACCACCAAG	25,947–25,924
LB	PED_LB_ID6	GGTACATTGCTTGTAGAGGGCTATAA	26,040–26,065

M: A or C

### RNA extraction

The total RNAs were extracted from 250 μl cell culture supernatants of PEDV, TGEV, and PRRSV, JEV, GV using a RNA extraction kit (ReliaPrep™ RNA Cell Miniprep System, Promega, USA), according to the manufacturer’s instructions.

### One-step RT-PCR

One-step RT-PCR was performed using AccessQuick™ RT-PCR System kits (Promega Corporation, WI, USA) as previously reported [12]. RT-PCR parameters included a reverse transcription step of 45 °C for 45 min and an incubation step of 94 °C for 2 min, 35 cycles at 94 °C for 30 s, 53 °C for 30 s, and 72 °C for 1 min, followed by the final extension at 72 °C for 10 min. The RT-PCR products were visualized by electrophoresis on a 1.5% agarose gel with ethidium bromide.

### RtF-RT-LAMP

The RtF-RT-LAMP reaction was conducted in a final reaction volume of 25 μl consisting of 2 μl RNA template, FIP and BIP primers (1.6 μM each), Loop F and Loop B primers (0.8 μM each), F3 and B3 primers (0.2 μM each), Isothermal Mastermixes (OptiGene, UK), 0.15 u of AMV reverse transcriptase (15 u/μl; Invitrogen, USA). Amplification reactions were performed at 63 °C for 40 min (with fluorescence detection followed by melt curve analysis from 90 to 70 °C at 0.05 °C/s), and then heated at a start temperature of 98 °C and end temperature of 80 °C for 10 min with a ramp rate of 0.05 °C/sec to terminate the reactions using Genie® III (OptiGene, UK). The fluorescence of the reaction was measured in real time, verifying the start of the amplification.

### Specificity of RtF-RT-LAMP

PEDV (adapted strain NK94P6), PRRSV (vaccine strain MLV), TEGV (vaccine strain h5), JEV (vaccine strain HmLu-SC) and GV (vaccine strain HAL-KB) were used as templates for RtF-RT-LAMP to analyse the specificity of RtF-RT-LAMP. Sterile ddH_2_O was used as the negative control.

### Sensitivity analysis of the RtF-RT-LAMP

To evaluate the sensitivity of the RtF-RT-LAMP, PEDV-infected Vero cell cultures of two strains (NK94P6 and Fukuoka-1 Tr(−)) with defined median tissue culture infective dose (TCID_50_) was tenfold serial diluted with the supernatant of negative faecal samples. RNA was then extracted from 250 μl media of each dilution and used as a template for RtF-RT-LAMP and one-step RT-PCR as mentioned above.

### Real-time RT-PCR

The quantitative One-Step PrimeScript RT-PCR kit (Takara Bio, Japan) was used for the real-time RT-PCR to quantitate two PEDV field strains (PEDV S INDEL and Non-S INDEL field strains). A 198 bp DNA fragment of the N gene was amplified with the primer sets of forward primer (qN306-F) 5’-CGCAAAGACTGAACCCACTAAC-3′ and reverse primer (56R) 5’-TTGCCTCTGTTGTTACTTGGAGAT-3′. A TaqMan probe (ProbeN466–469) with the sequence of 5’-GCAGGAGTCGTGGTAATGGCAACA-3′ was labeled with the 5′-reporter dye 6-carboxyfluorescein (FAM) and the 3′-quencher BHQ3. Real-time RT-PCR was carried out in a 20 μl reaction containing 2 μl of RNA template, 10 μl of 2X One Step RT-PCR Buffer III, 0.4 μl of TaKaRa Ex Taq HS, 0.4 μl of both forward and reverse primer, 0.8 μl of Probe, 0.4 μl of ROX Reference Dye and 5.6 μl of RNase free water. The reactions were performed using a StepOnePlus™ Real-Time PCR System (Amplified Biosystems, USA) under the following conditions: initial reverse transcription at 42 °C for 5 min, followed by initial denaturation at 95 °C for 10 s, 40 cycles of denaturation at 95 °C for 5 s, and annealing and extension at 60 °C for 30 s. The results of amplification were analyzed by StepOne Software v2.3 (Amplified Biosystems). Tenfold serial dilutions of the transcripts were prepared at concentrations of 8.97 × 10^7^ to 8.97 × 10^2^ copies of PEDV per 1 μl volume that were used for obtaining the standard curves.

### Detection of PEDV in clinical samples

A total of 99 faecal pig samples were collected from pig farms in Japan including 50 PED positive samples (from Kagoshima, Miyazaki, Aomori, Aichi prefectures) that were collected from December 2013 to August 2017 and 49 negative samples from a PED negative farm (Sumiyoshi farm, Miyazaki prefecture). These positive samples are classical, emerging Non-S INDEL, S INDEL, and S1 NTD-del PEDV variants and some positive samples are mixed infection of emerging non-S INDEL and S1 NTD-del PEDV variants. Faecal samples were prepared as a 10% (*w*/*v*) suspension in PBS (pH 7.2) and centrifuged at 2300 x g at 4 °C for 10 min. A 250-μl aliquot of supernatant was used for RNA extraction (ReliaPrep™ RNA Cell Miniprep System, Promega, USA). RNA was used as a template for detection of PEDV by one-step RT-PCR and RtF-RT-LAMP.

### Pooled samples

The RtF-RT-LAMP assay was used to determine efficiency in pooled stool samples for future application in large-scaled epidemiological surveys. We determined the sensitivity of the RT-LAMP assay in pooled faecal samples to calculate the possible sample sizes that can be applied to the pooled technique. Each PEDV positive sample was pooled with PEDV negative samples in different pooling ratios including 1:4, 1:9, 1:14, 1:19, 1:24, 1:29, 1:34, 1:39, 1:44, and 1:49. A 50-μl aliquot from individual positive or negative samples was transferred to a new tube and carefully votexed. Then, 250 μl in each pooling ratio was used for RNA extraction (ReliaPrep™ RNA Cell Miniprep System, Promega, USA). RNA was used as a template for RtF-RT-LAMP.

## Results

### Specificity of the RtF-RT-LAMP assay

**Fig. 1:**
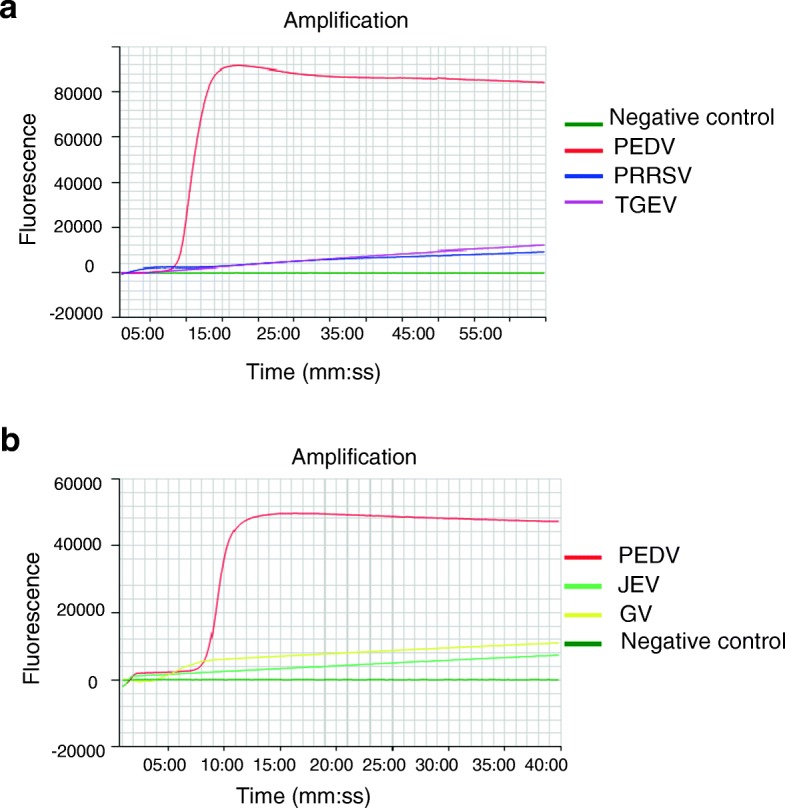
Specificity of the RtF-RT-LAMP assay for detecting the PEDV M gene. RNA of PEDV, PRRSV and TGEV (**a**); PEDV, JEV and GV (**b**) were used as templates for the RtF-RT-LAMP assay performed at 63 °C for 60 min

The PEDV NK94P6 strain and other related porcine viruses (PRRSV, TGEV, JEV, GV) were tested using the RtF-RT-LAMP assay to evaluate the specificity. Only PEDV was positive, and no LAMP products were detected in the reactions from other relevant porcine viruses or negative control used in this study (Fig. 1). The results indicated that the RtF-RT-LAMP assay was specific for PEDV and can be applied for distinguishing PEDV from other porcine viruses.

### Sensitivity of the RtF-RT-LAMP assay

To evaluate the sensitivity of the RtF-RT-LAMP assay, the detection limit was compared to the conventional one-step RT-PCR by amplifying ten-fold serial dilutions from the cell culture of two PEDV strains (NK94P6 and Fukuoka-1 Tr(−)). The detection limit of the one-step RT-PCR of the NK94P6 strain and Fukuoka-1 Tr(−) strain were 2.8 × 10^3^ TCID_50_/ml and 2 × 10^2^ TCID_50_/ml, while, the detection limit of the RtF-RT-LAMP assay was 2.8 × 10^1^ TCID_50_/ml and 2 × 10^0^ TCID_50_/ml, respectively (Tables 2 and 3). In addition, we used other two field strains that were PEDV S INDEL and Non-S INDEL strains to confirm the sensitivity of RtF-RT-LAMP (Additional files 2 and 3). This was much higher than that of the one-step RT-PCR. The sensitivity of the RtF-RT-LAMP assay was 100 times higher than that of one-step RT-PCR. Moreover, the real-time DNA fluorescence intensity from the reactions at all concentrations evaluated was high when the reactions were performed within 40 min. Therefore, the optimal reaction condition of the current RtF-RT-LAMP assay for PEDV was optimized for 40 min.

**Table 2:** Detection limits of one-step RT-PCR and RtF-RT-LAMP for the NK94P6 strain

TCID50	2.8 × 10^5^	2.8 × 10^4^	2.8 × 10^3^	2.8 × 10^2^	2.8 × 10^1^	2.8 × 10^0^	2.8 × 10^− 1^
One-step RT-PCR	+	+	+	–	–	–	
RtF-RT-LAMP (amplification time mm:ss)	+ (9:30)	+ (11:45)	+ (12:30)	+ (15:45)	+ (34:45)	–	–

+ Positive in duplicate

- Negative in duplicate

From 2.8 × 10^5^ to 2.8 × 10^− 1^: tenfold serial dilution of 2.8 × 10^6^ TCID50 PEDV NK94P6 strain

**Table 3:** Detection limits of one-step RT-PCR and RtF-RT-LAMP for the Fukuoka-1 Tr(−) strain

TCID50	2 × 10^3^	2 × 10^2^	2 × 10^1^	2 × 10^0^	2 × 10^− 1^	2 × 10^− 2^	2 × 10^− 3^
One-step RT-PCR	+	+	–	–	–	–	–
RtF-RT-LAMP (amplification time mm:ss)	+ 16:00	+ 18:15	+ 23:45	+ 35:45	±	–	–

+ Positive in duplicate

- Negative in duplicate

± One positive and one negative in duplicate

From 2 × 10^3^ to 2 × 10^− 3^: tenfold serial dilution of 2 × 10^4^ TCID50 PEDV Fukuoka-1 Tr(−) strain

### Detection of PEDV in clinical samples

To evaluate the sensitivity and specificity of the RtF-RT-LAMP assay to detect PEDV from clinical samples, one-step RT-PCR was used as the gold standard. A total of 99 clinical samples were tested by one-step RT-PCR that included 50 PED positive samples and 49 PED negative samples. All samples were tested by RtF-RT-LAMP assay. As shown in Tables 4, 49 PED negative samples were detected as negative and 50 PED positive samples were detected as positive by the RtF-RT-LAMP assay. No false negative or positive results were observed. Therefore, using one-step RT-PCR as the gold standard, the sensitivity and specificity of the RtF-RT-LAMP assay were 100%.

**Table 4:** Sensitivity and specificity of the RtF-RT-LAMP assay

		One-step RT-PCR		
		Number of positive samples	Number of negative samples	Total
RtF-RT-LAMP	Number of positive samples	50	0	50
	Number of negative samples	0	49	49
Total		50	49	99

### Pooled sample

**Fig. 2:**
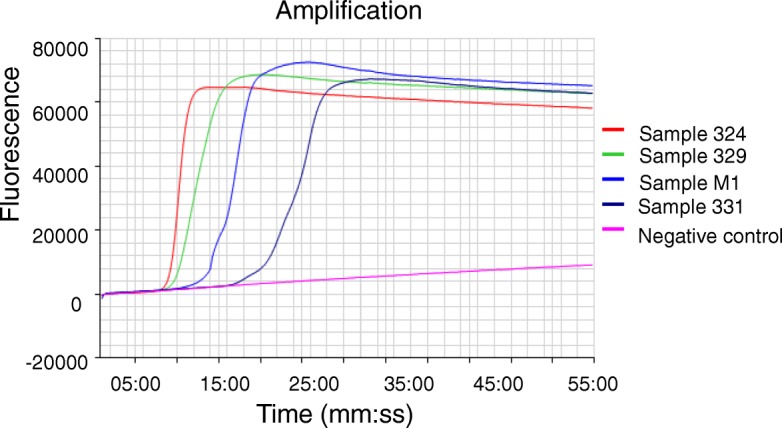
Positive faecal samples were used for estimating the pooled size. Four faecal samples were chosen from strongest positive to weakest positive that based on amplification time in the RtF-RT-LAMP assay and the intensity of electrophoresis bands of RT-PCR products

To estimate sample sizes for pooled faecal samples using the RtF-RT-LAMP assay, four positive samples were chosen from 50 positive samples based on amplification time in RT-LAMP and intensity of the electrophoresis band of RT-PCR products at different levels from the weakest positive to the strongest positive (Fig. 2). Sample 331 had the weakest electrophoresis band in RT-PCR. It was positive at the 25:45 min mark for amplification time, for which RT-LAMP can be positive until a pooling size of 15 samples. However, three other positive samples can be positive until a pooling size of 45 pooling or 50 samples (Table 5).

**Table 5:** Detection PEDV in pooled faecal samples by RtF-RT-LAMP

Pooled size	5	10	15	20	25	30	35	40	45	50
331	+	+	+	–	–	–	–	–	–	–
M1	+	+	+	+	+	+	+	+	+	–
329	+	+	+	+	+	+	+	+	+	+
324	+	+	+	+	+	+	+	+	+	+

Pooled size: Each PEDV positive sample was pooled with PEDV negative samples in different pooling ratios including 1:4, 1:9, 1:14, 1:19, 1:24, 1:29, 1:34, 1:39, 1:44, and 1:49

331, M1, 329, 324: Four positive samples were selected for pooling with a different number of negative samples

+: Positive by RtF-RT-LAMP

-: Negative by RtF-RT-LAMP

## Discussion

In this study, we successfully developed an RtF-RT-LAMP assay for detection of PEDV in pooled faecal samples as an economical protocol for detection of infected herd in surveillance or monitoring strategies of PED. A sensitive, specific, rapid, and simple RtF-RT-LAMP assay including loop primers from the M gene for PEDV detection was developed. The reaction condition of the RtF-RT-LAMP was optimized by selecting a primer set and simple incubation at 63 °C for 40 min. The sensitivity of the RtF-RT-LAMP assay for PEDV detection was at least 100 times higher than that of one-step RT-PCR. Particularly, by semi-quantitative analysis, the RtF-RT-LAMP assay was applied to identifying the size for pooled stool samples. Using the RtF-RT-LAMP assay, at least a pool of 15 individual faecal samples could be applied instead of testing individual samples for cost saving in PED surveillance or monitoring programmes.

The PEDV M protein is a highly conserved trans-membrane protein that is the most abundant envelope component [13, 14]. Two reports have shown that the developed RT-LAMP method for the PEDV M gene has a higher sensitivity than the RT-LAMP method developed for the N gene [15, 16]. The use of LAMP for detecting PEDV has been reported [15–17]. However, the previously described RT-LAMP assays for detecting PEDV were not monitored by real-time florescent devices. Furthermore, they only used four primers for the LAMP assay, and only the N gene was used for designing primers. Moreover, some mismatches were found between primers and template in the 3′-end of some primers that were used in the Gou et al. study [17]. Mismatches, especially within the 3′-end primer region, affect both the stability of the primer-template duplex and the efficiency with which the polymerase extends the primer, potentially leading to biased results or even failure [18, 19]. In this study, all primers, including loop primers, were designed from the highly conserved M gene of PEDV. To achieve maximum sensitivity of detection for PEDV, the primer set used in this study included both FIP primers (PED_FIP_ID1 and PED_FIP_ID1modified). PED_FIP_ID1 and PED_FIP_ID1modified shared nucleotide identity with approximately 95 and 5% available PEDV sequences in the GenBank, respectively. Importantly, the entire procedure of current RT-LAMP could be completed in a simple process within 50 min. Using RT-PCR as the gold standard, the sensitivity and specificity of the RtF-RT-LAMP assay reached 100%. Interestingly, the sensitivity and specificity of the RT-LAMP assay were not highlighted in the previously described RT-LAMP methods for PEDV detection [15–17]. Our results also indicate that the sensitivity of the RtF-RT-LAMP assay was much higher than that of the one-step RT-PCR. LAMP is a simple, rapid, specific and cost-effective nucleic acid amplification method because it provides high amplification efficiency with DNA being amplified 10^9^–10^10^ times in 15–60 min and use of 4 to 6 different primers to recognize 6 to 8 distinct regions on the target gene [9, 10]. In addition, LAMP is also applicable to RNA upon use of reverse transcriptase (RTase) together with DNA polymerase [20]. One study demonstrated that for PEDV detection, the sensitivity of qPCR was higher than that of RT-PCR [21]. RT-PCR and real-time RT-PCR techniques also demonstrate high specificity and sensitivity. However, these techniques require sophisticated and high-precision instruments (such as PCR and quantitative fluorescence PCR machines). Furthermore, the RT-PCR procedure is a time-consuming and complicated process. The RtF-RT-LAMP method showed distinct advantages with regards to detection time, as well as a simple process for rapid detection of PEDV.

Early identification of the infected herd through surveillance and monitoring strategies to enhance biosecurity measures is necessary to control PED. However, passive surveillance and individual testing could lead to important problems such being less effective as well as resulting in high cost and time commitments. Recently, a pooled sample technique has been developed and applied as a cost-efficient approach to surveillance or monitoring programs for pathogens such as Salmonella spp. in pigs and bovine viral diarrhoea virus in cattle [22, 23]. In this study, two pathogenically different PEDV strains and other two field strains were used to evaluate the sensitivity of the RtF-RT-LAMP assay. The RtF-RT-LAMP assay was much more sensitive than one-step RT-PCR even with different strains in TCID50 or copies. The optimal size for pooled stool samples was evaluated using a semi-quantitative method based on the amplification time in the RtF-RT-LAMP assay and intensity of the electrophoresis band for RT-PCR products. Even with the weakest positive electrophoresis band in one-step RT-PCR, in total 50 positive faecal samples was still positive in the RtF-RT-LAMP assay at a pooling size of 15, which was pooled from 14 individual negative faecal samples and one weak positive faecal sample. Furthermore, of the 50 PED positive samples, only two samples showed weak positive electrophoresis bands. Our pooled results indicate that a pool of at least 15 individual faecal samples can be applied using the RtF-RT-LAMP assay. In addition, the cost of one RT-PCR test was estimated based on the reagent cost, which was about 5 times more expensive than the RtF-RT-LAMP assay. In our study, testing pooled stool samples by RtF-RT-LAMP assay holds promise for surveillance and monitoring strategies. To our knowledge, this is the first study to provide evidence of the estimation of sample sizes for pooled stool samples for detecting PEDV using an accurate, simple, and timely RtF-RT-LAMP method. Our results will support the design and implementation of large-scaled epidemiological surveys as well as active surveillance or monitoring systems for effective control of PED. Further research will be required on a larger scale to confirm the effectiveness of the pooled sample protocol.

## Conclusions

In this study, the highly sensitive, specific, rapid, and simple RtF-RT-LAMP assay based on the M gene, using a mobile device for detection of PEDV in pooled stool of at least 15 samples was shown to be an economical diagnosis test for PEDV detection. Use of these methods will not only be feasible, but also serve as effective surveillance and monitoring strategies to control PED.

## Additional files

Additional file 1: RT-LAMP primers design for PEDV nucleotide detection. Nucleotide sequence alignments of M gene of seven PEDV strains. Representative M gene sequences in each strain are aligned with clustalW. Sequence data of designing primers for RT-LAMP in this study (KT323979.1), the sequence used for RT-PCR (JX435310.1 and JN089738.1), the sequence of G1b S INDEL strain (KY619833.1), the sequence of G2b/Non S INDEL/North America strain (KY619838.1), the sequence of G2a/Non S INDEL/Asian strain (KJ960178.1), the sequence of NK96P4C6 G1a classical strain (KY619828). Primer recognition sites are indicated with primer names. (DOCX 28 kb)

Additional file 2: Detection limits of one-step RT-PCR and RtF-RT-LAMP for PEDV S INDEL field strain**.** From 5.0 × 10^5^ to 5.0 × 10^0^: tenfold serial dilution of 5.0 × 10^6^ copies PEDV S INDEL field strain. (DOCX 14 kb)

Additional file 3: Detection limits of one-step RT-PCR and RtF-RT-LAMP for the PEDV Non-S INDEL field strain. From 1.5 × 10^6^ to 1.5 × 10^0^: tenfold serial dilution of 1.5 × 10^7^ copies PEDV Non-S INDEL field strain. (DOCX 14 kb)

## Abbreviations

EMEM: Eagle’s minimal essential medium
FBS: Foetal bovine serum
GV: Getal virus
JEV: Japanese Encephalitis virus
PED: Porcine epidemic diarrhoea
PEDV: Porcine epidemic diarrhoea virus
PRRSV: Porcine reproductive and respiratory syndrome virus
RtF-RT-LAMP: Real-time fluorescent reverse transcription loop-mediated isothermal amplification
RT-PCR: Reverse transcription polymerase chain reaction
TCID: Tissue culture infective dose
TGEV: Transmissible gastroenteritis coronavirus
TPB: Tryptose phosphate broth
