# Supplementary material for: Development of pooled testing system for porcine epidemic diarrhoea using real-time fluorescent reverse-transcription loop-mediated isothermal amplification assay
Source: BMC Vet Res. 2018 May 29;14:172. doi: 10.1186/s12917-018-1498-9 (PMC5975689; doi:10.1186/s12917-018-1498-9)
Supplement: Supplementary file 1 — RT-LAMP primers design for PEDV nucleotide detection. Nucleotide sequence alignments of M gene of seven PEDV strains. Representative M gene sequences in each strain are aligned with clustalW. Sequence data of designing primers for RT-LAMP in this study (KT323979.1), the sequence used for RT-PCR (JX435310.1 and JN089738.1), the sequence of G1b S INDEL strain (KY619833.1), the sequence of G2b/Non S INDEL/North America strain (KY619838.1), the sequence of G2a/Non S INDEL/Asian strain (KJ960178.1), the sequence of NK96P4C6 G1a classical strain (KY619828). Primer recognition sites are indicated with primer names. (DOCX 28 kb) [file 12917_2018_1498_MOESM1_ESM.docx]

**Additional file 1:** **RT-LAMP primers design for PEDV nucleotide detection**

60 120

KT323979.1: ATGTCTAACGGTTTTATTCCCGTTGATGAGGTGATTGAACACCTTAGAAACTGGAATTTCACATGGAATATCATACTGACGATACTACTTGTAGTGCTTCAGTATGGCCATTACAAGTAC

JX435310.1: ATGTCTAACGGTTCTATTCCCGTTGATGAGGTGATTCAACACCTTAGAAACTGGAATTTCACATGGAATATCATACTGACGATACTACTTGTAGTGCTTCAGTATGGCCATTACAAGTAC

KY619833.1: ATGTCTAACGGTTCTATTCCCGTTGATGAGGTGATTCAACACCTTAGAAACTGGAATTTCACATGGAATATCATACTGACGATACTACTTGTAGTGCTTCAGTATGGCCATTACAAGTAC

KY619838.1: ATGTCTAACGGTTCTATTCCCGTTGATGAGGTGATTCAACACCTTAGAAACTGGAATTTCACATGGAATATCATACTGACGATACTACTTGTAGTGCTTCAGTATGGCCATTACAAGTAC

KJ960178.1: ATGTCTAACGGTTCTATTCCCGTTGATGAGGTGATTGAACACCTTAGAAACTGGAATTTCACATGGAATATCATACTGACGATACTACTTGTAGTGCTTCAGTATGGCCATTACAAGTAC

JN089738.1: ATGTCTAACGGTTCTATTCCCGTTGATGAGGTGATTCAACACCTTAGAAACTGGAATTTCACGTGGAATATCATACTGACGATACTACTTGTAGTGCTTCAGTATGGCCATTACAAGTAC

KY619828 : ATGTCTAACGGTTTTATTCCCGTTGATGAGGTGGTTCAACACCTTAGAAACTGGAATTTTACATGGAATATCATACTGACGATACTACTTGTAGTGCTTCAGTATGGCCATTACAAGTAC

************* *******************.** ********************** **.*********************************************************

180 240

: TCTGTGTTCTTGTATGGTGTCAAGATGGCTATTCTATGGATACTTTGGCCTCTTGTGTTGGCACTGTCACTTTTTGATGCATGGGCTAGCTTCCAGGTCAACTGGGTCTTTTTCGCTTTC

: TCTGCGTTCTTGTATGGTGTCAAGATGGCTATTCTATGGATACTTTGGCCTCTTGTGTTAGCACTGTCACTTTTTGATGCATGGGCTAGCTTTCAGGTCAATTGGGTCTTTTTTGCTTTC

: TCTGCGTTCTTGTATGGTGTCAAGATGGCTATTCTATGGATACTTTGGCCTCTTGTGTTAGCACTGTCACTTTTTGATGCATGGGCTAGCTTTCAGGTCAATTGGGTCTTTTTTGCTTTC

: TCTGCGTTCTTGTATGGTGTCAAGATGGCTATTCTATGGATACTTTGGCCTCTTGTGTTAGCACTGTCACTTTTTGATGCATGGGCTAGCTTTCAGGTCAATTGGGTCTTTTTTGCTTTC

: TCTGCGTTCTTGTATGGTGTCAAGATGGCTATTCTATGGATACTTTGGCCTCTTGTGTTGGCACTTTCACTTTTTGATGCATGGGCTAGCTTTCAGGTCAACTGGGTCTTTTTTGCTTTC

: TCTGCGTTCTTGTATGGTGTCAAGATGGCTATTCTATGGATACTTTGGCCTCTTGTGTTAGCACTGTCACTTTTTGATGCATGGGCTAGCTTTCAGGTCAATTGGGTCTTTTTTGCTTTC

: TCTGCGTTCTTGTATGGTGTCAAGATGGCTATTCTATGGATACTCTGGCCTCTTGTGTTGGCACTGTCACTTTTTGATGCATGGGCTAGCTTCCAGGTCAATTGGGTCTTTTTTGCTTTC

**** *************************************** **************.***** ************************** ******** *********** ******

**PED-F3** 300 **PED-F2 PED-LF** 360

: AGCATCCTTATGGCTTGCATCACTCTTATGCTGTGGATAATGTATTTTGTCAATAGCATTCGGTTGTGGCGCAGGACACATTCTTGGTGGTCTTTCAATCCTGAAACTGACGCGCTTCTC

: AGCATCCTTATGGCTTGCATCACTCTTATGCTGTGGATAATGTACTTTGTCAATAGCATTCGGTTGTGGCGCAGGACACATTCTTGGTGGTCTTTCAATCCTGAAACAGACGCGCTTCTC

: AGCATCCTTATGGCTTGCATCACTCTTATGCTGTGGATAATGTACTTTGTCAATAGCATTCGGTTGTGGCGCAGGACACATTCTTGGTGGTCTTTCAACCCTGAAACAGACGCGCTTCTC

: AGCATCCTTATGGCTTGCATCACTCTTATGCTGTGGATAATGTACTTTGTTAATAGCATTCGGTTGTGGCGCAGGACACATTCTTGGTGGTCTTTCAATCCTGAAACAGACGCGCTTCTC

: AGCATCCTTATGGCTTGCATCACTCTTATGCTGTGGATAATGTACTTTGTCAATAGCATTCGGTTGTGGCGCAGGACACATTCTTGGTGGTCTTTCAATCCTGAAACAGACGCGCTTCTC

: AGCATCCTTATGGCTTGCATCACTCTTATGCTGTGGATAATGTACTTTGTCAATAGCATTCGGTTGTGGCGCAGGACACATTCTTGGTGGTCTTTCAATCCTGAAACAGACGCGCTTCTC

: AGCATCCTTATGGCTTGCATCACTCTTATGCTGTGGATAATGTATTTTGTCAATAGCATTCGGTTGTGGCGCAGGACACATTCTTGGTGGTCTTTCAATCCTGAAACTGACGCGCTTCTC

: ******************************************** ***** *********************************************** ********:************

**PED-F1c** **PED-B1c** 420 **PED-LB** **PED-B2** 480

: ACTACTTCTGTGATGGGCCGACAGGTCTGCATTCCAGTGCTTGGAGCACCAACTGGTGTAACGCTAACACTCCTTAGTGGTACATTGTTTGTAGAGGGCTATAAGGTTGCTACTGGCGTA

: ACTACTTCTGTGATGGGCCGACAGGTCTGCATTCCAGTGCTTGGAGCACCAACTGGTGTAACGCTAACACTCCTTAGTGGTACATTGCTTGTAGAGGGCTATAAGGTTGCTACTGGCGTA

: ACTACTTCTGTGATGGGCCGACAGGTCTGCATTCCAGTGCTTGGAGCACCAACTGGTGTAACGCTAACACTCCTTAGTGGTACATTGCTTGTAGAGGGCTATAAGGTTGCTACTGGCGTA

: ACTACTTCTGTGATGGGCCGACAGGTCTGCATTCCAGTGCTTGGAGCACCAACTGGTGTAACGCTAACACTCCTTAGTGGTACATTGCTTGTAGAGGGCTATAAGGTTGCTACTGGCGTA

: ACTACTTCTGTGATGGGCCGACAGGTCTGCATTCCAGTGCTTGGAGCACCAACTGGTGTAACGCTAACACTCCTTAGTGGTACATTGCTTGTAGAGGGCTATAAGGTTGCTACTGGCGTA

: ACTACTTCTGTGATGGGCCGACAGGTCTGCATTCCAGTGCTTGGAGCACCAACTGGTGTAACGCTAACACTCCTTAGTGGTACATTGCTTGTAGAGGGCTATAAGGTTGCTACTGGCGTA

: ACTACTTCTGTGATGGGCCGACAGGTCTGCATTCCTGTGCTTGGGGCACCAACTGGTGTAACGCTAACACTCCTTAGTGGTACATTGTTTGTAGAGGGCTATAAGGTTGCTACTGGCGTA

: ***********************************:********.****************************************** ********************************

**PED-B3** 540

: CAGGTAAGTCAATTGCCTGATTTCGTCACAGTCGCCAAGGCCACTACAACAATTGTCTATGGACGTGTTGGTCGTTCAGTCAATGCTTCATCTGGCACTGGTTGGGCTTTCTATGTCCGG

: CAGGTAAGTCAATTACCTAATTTCGTCACAGTCGCCAAGGCCACTACAACAATTGTCTACGGACGTGTTGGTCGTTCAGTCAATGCTTCATCTGGCACTGGTTGGGCTTTCTATGTCCGG

: CAGGTAAGTCAATTACCTAATTTCGTCACAGTCGCCAAGGCCACTACAACAATTGTCTACGGACGTGTTGGTCGTTCAGTCAATGCTTCATCTGGCACTGGTTGGGCTTTCTATGTCCGG

: CAGGTAAGTCAATTACCTAATTTCGTCACAGTCGCCAAGGCCACTACAACAATTGTCTACGGACGTGTTGGTCGTTCAGTCAATGCTTCATCTGGCACTGGTTGGGCTTTCTATGTCCGG

: CAGGTAAGTCAATTACCTAATTTCGTCACAGTCGCCAAGGCCACTACAACAATTGTCTATGGACGTGTTGGTCGTTCAGTCAATGCTTCATCTAGCACTGGTTGGGCTTTCTATGTCCGG

: CAGGTAAGTCAATTACCTAACTTCGTCACAGTCGCCAAGGCCACTACAACAATTGTCTACGGACGTGTTGGTCGTTCAGTCAATGCTTCATCTGGCACTGGTTGGGCTTTCTATGTCCGG

: CAGGTAAGTCAATTACCTAATTTCGTCACAGTCGCCAAGGCCACTACAACAATTGTCTATGGACGTGTTGGTCGTTCAGTCAATGCTTCATCTGGCACTGGTTGGGCTTTCTATGTCCGG

: **************.***.* ************************************** *********************************.**************************

: TCAAAACACGGCGACTACTCAGCTGTGAGTAATCCGAGTGCGGTTCTCACAGATAGTGAGAAAGTGCTTCATTTAGTCTAA

: TCCAAACACGGCGACTACTCAGCTGTGAGTAATCCGAGTTCGGTTCTCACAGATAGTGAGAAAGTGCTTCATTTAGTCTAA

: TCCAAACACGGCGACTACTCAGCTGTGAGTAATCCGAGTTCGGTTCTCACAGATAGTGAGAAAGTGCTTCATTTAGTCTAA

: TCCAAACACGGCGACTACTCAGCTGTGAGTAATCCGAGTTCGGTTCTCACAGATAGTGAGAAAGTGCTTCATTTAGTCTAA

: TCAAAACACGGCGACTACTCAGCTGTGAGTAATCCGAGTGCGGTTCTCACAGATAGTGAGAAAGTGCTTCATTTAGTCTAA

: TCCAAACACGGCGACTACTCAGCTGTGAGTAATCCGAGTTCGGTTCTCACAGATAGTGAGAAAGTGCTTCATTTAGTCTAA

: TCTAAACACGGCGACTACTCAGCTGTGAGTAATCCGAGTGCGGTCCTCACAGATAGTGAGAAAGTGCTTCATTTAGTCTAA

** ************************************ **** ************************************

Nucleotide sequence alignments of M gene of seven PEDV strains. Representative M gene sequences in each strain are aligned with clustalW. Sequence data of designing primers for RT-LAMP in this study (KT323979.1), the sequence used for RT-PCR (JX435310.1 and JN089738.1), the sequence of G1b S INDEL strain (KY619833.1), the sequence of G2b/Non S INDEL/North America strain (KY619838.1), the sequence of G2a/Non S INDEL/Asian strain (KJ960178.1), the sequence of NK96P4C6 G1a classical strain ([KY619828](https://www.ncbi.nlm.nih.gov/nuccore/KY619828)). Primer recognition sites are indicated with primer names.

*: Indicates conserved nucleotides
