# Supplementary material for: Development of pooled testing system for porcine epidemic diarrhoea using real-time fluorescent reverse-transcription loop-mediated isothermal amplification assay
Source: BMC Vet Res. 2018 May 29;14:172. doi: 10.1186/s12917-018-1498-9 (PMC5975689; doi:10.1186/s12917-018-1498-9)
Supplement: Supplementary file 2 — Detection limits of one-step RT-PCR and RtF-RT-LAMP for PEDV S INDEL field strain. From 5.0 × 10^5^ to 5.0 × 100: tenfold serial dilution of 5.0 × 10^6^ copies PEDV S INDEL field strain. (DOCX 14 kb) [file 12917_2018_1498_MOESM2_ESM.docx]

**Additional file 2:** Detection limits of one-step RT-PCR and RtF-RT-LAMP for PEDV S INDEL field strain

| Copies | 5.0x10^5^ | 5.0x10^4^ | 5.0x10^3^ | 5.0x10^2^ | 5.0x10^1^ | 5.0x10^0^ |
| --- | --- | --- | --- | --- | --- | --- |
| One-step RT-PCR | + | + | - | - | - | - |
| RtF-RT-LAMP (Amplification time mm:ss) | +  (10:15) | +  (13:15) | +  (18:30) | +  (28:15) | - | - |

+ Positive in duplicate

- Negative in duplicate

From 5.0 x 10^5^ to 5.0 x 10^0^: tenfold serial dilution of 5.0x10^6^ copies PEDV S INDEL field strain
