# Supplementary material for: Development of pooled testing system for porcine epidemic diarrhoea using real-time fluorescent reverse-transcription loop-mediated isothermal amplification assay
Source: BMC Vet Res. 2018 May 29;14:172. doi: 10.1186/s12917-018-1498-9 (PMC5975689; doi:10.1186/s12917-018-1498-9)
Supplement: Supplementary file 3 — Detection limits of one-step RT-PCR and RtF-RT-LAMP for the PEDV Non-S INDEL field strain. From 1.5 × 10^6^ to 1.5 × 100: tenfold serial dilution of 1.5 × 10^7^ copies PEDV Non-S INDEL field strain. (DOCX 14 kb) [file 12917_2018_1498_MOESM3_ESM.docx]

**Additional file 3:** Detection limits of one-step RT-PCR and RtF-RT-LAMP for the PEDV Non-S INDEL field strain

| Copies | 1.5x10^6^ | 1.5x10^5^ | 1.5x10^4^ | 1.5x10^3^ | 1.5x10^2^ | 1.5x10^1^ | 1.5x10^0^ |
| --- | --- | --- | --- | --- | --- | --- | --- |
| RT-PCR | + | + | + | + | - | - | - |
| RT-LAMP (Amplification time  mm:ss) | +  (7:30) | +  (8:30) | +  (9:45) | +  (10:15) | +  (12:45) | +  (20:45) | - |

+ Positive in duplicate

- Negative in duplicate

From 1.5 x 10^6^ to 1.5 x 10^0^: tenfold serial dilution of 1.5x10^7^ copies PEDV Non-S INDEL field strain
